# DUSP6 inhibitor (E/Z)-BCI hydrochloride stimulates glucose clearance and adipose lipolysis in diet-induced obese mice

**DOI:** 10.1016/j.gendis.2025.101671

**Published:** 2025-05-08

**Authors:** Xiaohua Huang, Wei Lu, Dandan Jiang, Zhengfeng Fang, Bin Feng

**Affiliations:** a Meat Processing Key Laboratory of Sichuan Province, College of Food and Biological Engineering, Chengdu University, Chengdu, Sichuan 610106, China; b Animal Nutrition Institute, Sichuan Agricultural University, Chengdu, Sichuan 611130, China; c Key Laboratory of Animal Disease-Resistant Nutrition of Ministry of Education, Sichuan Agricultural University, Chengdu, Sichuan 611130, China; d Key Laboratory for Food Science and Human Health, College of Food Science, Sichuan Agricultural University, Ya'an, Sichuan 625014, China

Obesity-related metabolism diseases, such as obesity, insulin resistance, hyperglycemia and hyperlipemia, have emerged as important chronic disease.^1^^,^^2^ BCI hydrochloride (BCI), a small-molecule inhibitor that reportedly can effective to decrease the dual-specificity phosphatases (DUSP) 1 and 6 phosphatase activity,^3^ is linked to several biological processes, such as preventing tumor development and macrophage inflammation.^4^^,^^5^ This study was performed to investigate the role and mechanism of BCI treatment on glucose and lipid metabolism in mice. Results showed that BCI markedly reduced epididymal adipose tissue weight in diet-induced obese (DIO) mice. In addition, BCI-treated obese mice showed better glucose clearance and less hyperglycemia than control mice. Furthermore, the gene expression of lipolytic genes in the epididymal adipose tissue were higher in BCI treatment mice than those in control mice.

C57BL/6J male mice were fed with high-fat diet from 5-week to 16-week of age, then were intraperitoneally injected with vehicle or BCI. Detailed informations are provided in Supplementary Materials and Methods, and Table S1. The food intake and body weight of BCI-treated mice were similar to those control mice (Fig. S1A, B). The weight of epididymal adipose tissue was much lower in the BCI-treated mice than that of the control mice, while the weights of perirenal adipose, subcutaneous adipose and liver tissues were unchanged by BCI treatment (Fig. 1A). These data indicated that BCI treatment could reduce epididymal adipose tissue weight in DIO mice.

**Figure 1:**
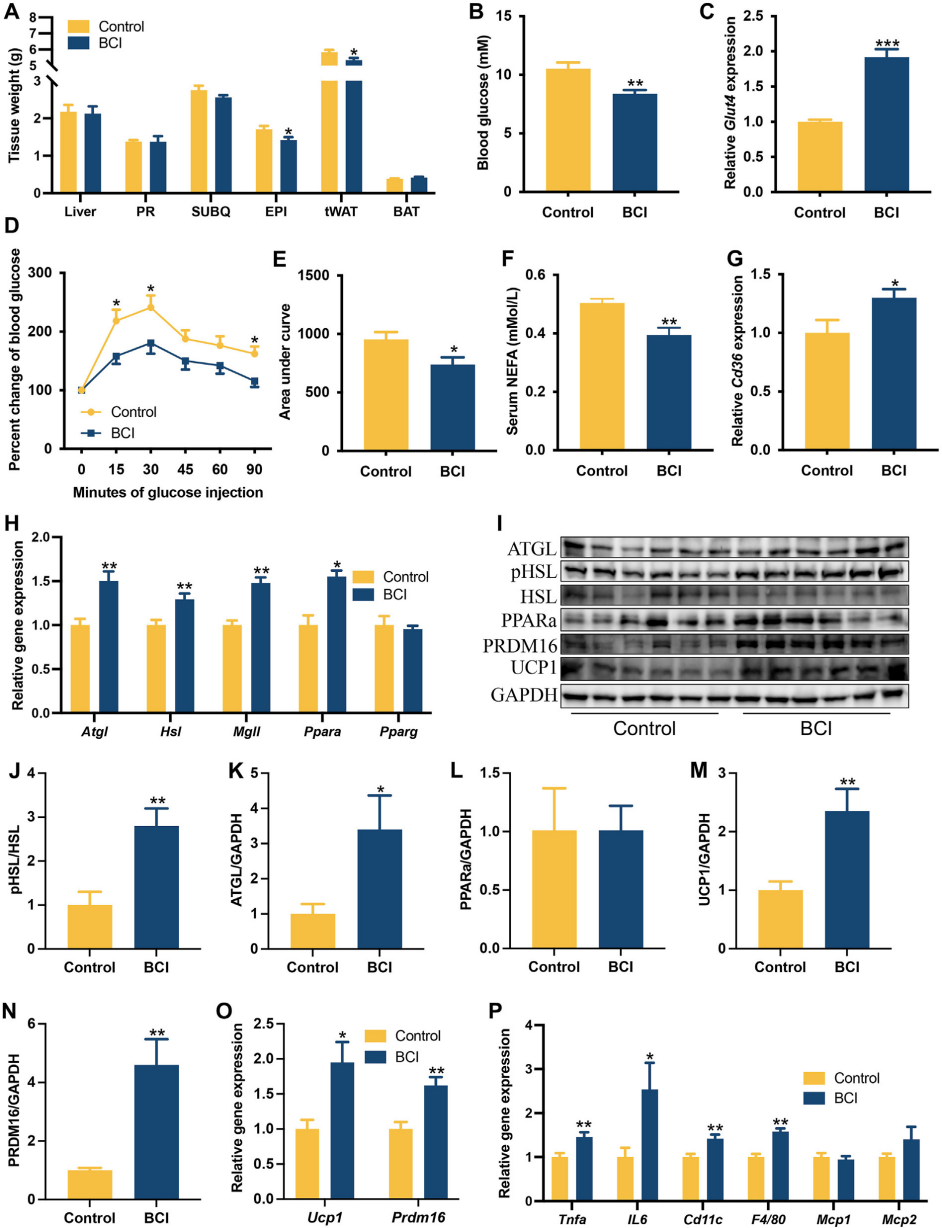
BCI protected diet-induced obese mice from hyperglycemia. C57BL/6J mice were fed with a high-fat diet for 16 weeks, followed by intraperitoneally injection with 0.5 mg/kg BCI or vehicle daily for 4 weeks, and then were sacrificed under fed state for serum and tissue collection (*N* = 6 per group). **(A)** Tissue weight of the mice. **(B)** Blood glucose level at harvest. **(C)** The expression level of *Glut4* in the epididymal adipose tissue. **(D, E)** Glucose tolerance test study (*N* = 6 for each group). **(F)** Serum content of NEFA. **(G)** The expression level of *Cd36* in the epididymal adipose tissue. **(H)** Expression levels of lipolytic genes in the epididymal adipose tissue. **(I–N)** Protein levels of ATGL, PPARa, PRDM16 and UCP1, and phosphorylation level of HSL in the epididymal adipose tissue. Expression levels of thermogenic **(O)** and inflammatory **(P)** genes in the epididymal adipose tissue. Data are expressed as Mean ± SE. ∗*P* < 0.05, ∗∗*P* < 0.01, ∗∗∗*P* < 0.001 as compared to control. PR, perirenal adipose tissue; SUBQ, subcutaneous adipose tissue; EPI, epididymal adipose tissue; tWAT, total white adipose tissue; BAT, brown adipose tissue.

We then investigated the role of BCI treatment on glucose and lipid metabolism in DIO mice. Results showed that BCI treated mice had lower blood glucose level than the control mice under fed state (Fig. 1B). Besides, the mRNA levels of glucose transporter 4 (*Glut4*), the main transporter for glucose uptake in adipocyte, was increased in the epididymal fat of BCI-treated mice, as compared with control group (Fig. 1C). Moreover, glucose tolerance test (GTT) study showed that BCI-treated mice had lower blood glucose levels 15, 30 and 90 min after exogenous glucose administration than control mice did, which meaned higher glucose clearance rate (Fig. 1D, E). However, insulin tolerance test (ITT) was not changed by BCI treatment, as compared to control mice (Fig. S1C, D). These data indicated that BCI could improve hyperglycemia and systemic glucose clearance rate by stimulating glucose uptake of adipose tissue in obese mice.

Serum lipid profiles were further investigated, which indicated that the serum NEFA concentration was lower in the BCI-treated mice than that of the control mice under high-fat diet condition (Fig. 1F). Furthermore, the expression of fatty acid translocase (*Cd36*), a fatty acid transporter which helps cells uptake free fatty acids, was increased in the epididymal adipose tissue by BCI treatment compared with control (Fig. 1G). However, serum concentrations of triglycerides (TAG), total cholesterol (TC), low-density lipoprotein cholesterol (LDL-C) and high-density lipoprotein cholesterol (HDL-C) were unchanged by BCI treatment compared with the control (Fig. S1E–H). These data suggest that BCI might reduce circulation NEFA concentration by increasing fatty acids uptake of adipocytes at least in eWAT.

The expression lipogenic and lipolytic genes were then investigated in epididymal adipose tissue. Results showed that BCI administration increased the expression of lipid catabolic genes, including adipose triglyceride lipase (*Atgl*), hormone-sensitive lipase (*Hsl*), monoglyceride lipase (*Mgll*) and peroxisome proliferator-activated receptor alpha (*Ppara*) in the epididymal adipose tissue compared with the control treatment, although the expression of peroxisome proliferator proliferator-activated receptor gamma (*Pparg*) was unchanged (Fig. 1H). Furthermore, the protein level of ATGL and phosphorylation level of HSL in epididymal adipose tissue of BCI-treated mice were higher than those of the control mice, although no change was observed in the protein level of PPARa between the two groups (Fig. 1I–L). However, the mRNA levels of lipogenic genes, including fatty acids synthetase (*Fasn*), stearoyl-CoA desaturase 1 (*Scd1*), acetyl-CoA carboxylase1 and 2 (*Acc1* and *Acc2*) and sterol regulatory element binding transcription factor 1 (*Srebf1*), and triglyceride synthetic gene diacylglycerol acyltransferase 1 (*Dgat1*) were not change by BCI treatment in epididymal adipose tissue (Fig. S1I). In addition, the mRNA and protein levels of thermogenic genes, uncoupling protein 1 (UCP1) and PR domain zinc finger protein 16 (PRDM16) were significantly increased in the epididymal adipose tissue of BCI-treated mice (Fig. 1I–O), although no change was observed in the mRNA level of peroxisome proliferator-activated receptor gamma coactivator 1 alpha (*Pgc1a*) between the two groups (Fig. S1J). These data suggest that BCI might be not only a potential adipocyte lipolysis stimulator, but also can induce adipocyte beige.

Obesity is always companied with macrophage infiltration and inflammation in adipose tissue. Thus, the mRNA levels of cytokine and macrophage marker genes in the epididymal adipose tissue were investigated. Results indicated that the expression of macrophage marker genes *Cd11c* and *F4/80,* and inflamatory cytokine genes tumor necrotic factor alpha (*Tnfa*) and interleukin 6 (*Il6*) were increased in the epididymal adipose tissue of BCI-treated mice as compared to those in the control mice (Fig. 1P). However, the mRNA levels of chemokine genes *Mcp1* and *Mcp2* were not change in epididymal adipose tissue by BCI treatment (Fig. 1P). These data suggest that BCI might induce macrophage infiltration and inflammation in adipose tissue.

The role of BCI on glucose and lipid metabolism under normal physiological condition was also investigated in lean mice. Results showed that BCI administration decreased blood glucose level under fed state, but did not change food intake, body weight, tissue weight, or serum contents of TAG, NEFA, TC, HDL-C or LDL-C (Fig. S2A–I).

In summary, the current study revealed that BCI could stimulate glucose clearance and alleviate hyperglycemia likely by promoting glucose uptake and lipolysis in visceral adipose tissue of diet-induced obesity. Thus, DUSP6 inhibitor BCI might be a therapeutic molecular for obesity related hyperglycemia.

## CRediT authorship contribution statement

**Xiaohua Huang:** Writing – original draft, Methodology, Investigation. **Wei Lu:** Investigation. **Dandan Jiang:** Investigation. **Zhengfeng Fang:** Investigation. **Bin Feng:** Writing – review & editing, Project administration, Methodology, Investigation, Funding acquisition.

## Funding

This study was supported by the 10.13039/100014717National Natural Science Foundation of China (32272893).

## Conflict of interests

The authors declare no conflict of interests.

## References

[1] Wong V.W., Ekstedt M., Wong G.L., Hagstrom H. (2023). Changing epidemiology, global trends and implications for outcomes of NAFLD. J Hepatol.

[2] Pillon N.J., Loos R.J.F., Marshall S.M., Zierath J.R. (2021). Metabolic consequences of obesity and type 2 diabetes: balancing genes and environment for personalized care. Cell.

[3] Benito-Leon M., Gil-Redondo J.C., Perez-Sen R., Delicado E.G., Ortega F., Gomez-Villafuertes R. (2022). BCI, an inhibitor of the DUSP1 and DUSP6 dual specificity phosphatases, enhances P2X7 receptor expression in neuroblastoma cells. Front Cell Dev Biol.

[4] Molina G., Vogt A., Bakan A. (2009). Zebrafish chemical screening reveals an inhibitor of Dusp6 that expands cardiac cell lineages. Nat Chem Biol.

[5] Momeny M., Tienhaara M., Sharma M. (2024). DUSP6 inhibition overcomes neuregulin/HER3-driven therapy tolerance in HER2+ breast cancer. EMBO Mol Med.

