# Supplementary material for: DUSP6 inhibitor (E/Z)-BCI hydrochloride stimulates glucose clearance and adipose lipolysis in diet-induced obese mice
Source: Genes Dis. 2025 May 8;13(2):101671. doi: 10.1016/j.gendis.2025.101671 (PMC12664611; doi:10.1016/j.gendis.2025.101671)
Supplement: Multimedia component 1 [file mmc1.docx]

**Materials and Methods**

**Animal models**

Animal protocols were reviewed and approved by the Animal Care and Use Committee of Sichuan Agricultural University. All animal studies were performed under the guide of Use and Care of Laboratory Animals. 3-week-old C57BL/6J mice were purchased from GenPharmatech (Nanjing, China). The mice were housed in a pathogen-free room at 22℃ and 60% of stable temperature and humidity. After one-week adaptation period, the mice were randomly grouped based on body weight and were fed with a normal chow diet (NCD, D12450J, Research Diet) or a high-fat diet (HFD, 60% kcal from fat, D12492, Research Diet) with free access to water. After 16 weeks of feeding, the mice were randomly divided into 2 groups and injected intraperitoneally with 0.5 mg/kg BCI (B4313, Sigma, Shanghai, China) or vehicle daily for 4 weeks. Food intake and body weight were measured. At 24 weeks of age, mice were accessed for blood glucose measurement with glucose test strip (Beijing Yicheng) under fed status, and then were euthanized. Serum samples was collected for further analysis. The liver, perirenal, epididymal and subcutaneous adipose tissue were collected, weighed, and stored at -80℃ for subsequent analyses.

**Insulin tolerance test (ITT)**

After 6 hours of fasting, mice were administered with insulin (Novonordisk, Beijing, China) intraperitoneally at the doses of 0.5 IU/kg (lean mice) or 1 IU/kg (DIO mice). Blood glucose levels were measured at 0, 15, 30, 45, 60, and 90 minutes after the injection of insulin, as previously described.^1^

**Glucose tolerance test (GTT)**

After overnight fasting, mice were intraperitoneally injected with 0.5 g/kg (lean mice) or 1 g/kg (DIO mice) glucose (Sigma, G7021, Shanghai, China). Blood glucose levels were measured at 0, 15, 30, 45, 60, and 90 minutes after glucose injection, as previously described.^2^

**RNA extraction and real-time PCR (qRT-PCR)**

RNA extraction and real-time PCR procedures were conducted following previously report.^2^ Briefly, the adipose tissue was subjected to total RNA extraction using TRIzol reagent (Sigma) according to the manufacturer’s instruction. The quality of the extracted total RNA was analyzed using agarose gel electrophoresis, while the concentration of RNA was determined using a spectrophotometer (NanoDrop 2000, Thermo Scientific). Complementary DNA (cDNA) synthesis was accomplished using a reverse-transcription PCR kit (RR047A, Takara, Dalian, China). Real-time PCR analysis was performed on a high-quality PCR machine (7900HT, ABI, Carlsbad, CA, USA) utilizing Power SYBR Green RT-PCR reagents (4367659, Thermo Fisher Scientific). The PCR reaction was set as a thermal cycling program of 95℃ for 10 minutes for one cycle, followed by 40 cycles of denaturation at 95℃ for 15 seconds and annealing/extension at 60℃ for 1 minute. Gene expression levels were quantified using the 2^-ΔΔCt^ method, with beta-actin serving as the reference gene. The primer sequences are showed in Table S1.

**Western blot analysis**

Protein extraction procedures were conducted following previously report.^1^ Briefly, adipose tissue was homogenized in cell lysis buffer (Beyotime Biotechnology, Shanghai, China) supplemented with a protease inhibitor cocktail (4693116001, Roche, Mannheim, Germany) using a homogenizer. Subsequently, 30 μg of total protein was separated by SDS-PAGE polyacrylamide gel and transferred onto PVDF membranes for antibody blotting. ATGL (2138), pHSL (3891) and UCP1 (14670) antibodies were obtained from Cell Signaling Technology; HSL (sc-74489) and PPARα (sc-398394) antibody were the products of Santa Cruz (Shanghai, China); PRDM16 (PA5-20872) and GAPDH (abs132004) antibodies were obtained from Thermo Fisher Scientific (Shanghai, China) and Absin Biotechnology Company (Shanghai, China), respectively. After thorough washing, the membranes were incubated with suitable horseradish peroxidase-linked secondary antibodies (#7074 and #7076, Cell Signaling Technology) for 1 hour. Following additional washing steps, protein signals were detected using ECL western blotting detection reagent (1705060, BioRad) on a Molecular Imager ChemiDoc XRS+ System (BioRad).

**Statistical Analysis**

The data were analyzed using SAS 9.3 software (SAS Institute Inc., Cary, NC, USA). Firstly, the normality and homogeneity of variances of the data were assessed through univariate test. For normally distributed data, the independent t-test was employed to compare differences between two groups, while non-Gaussian and heterogeneous data were analyzed using non-parametric methods. To analyze the statistical difference of ITT and GTT, two-way repeated measures ANOVA was applied. The results were presented as mean ± SE, and statistical significance was determined at *P*-value less than 0.05.

**References**

1 Huang X, Jin L, Fang Z, et al. Identification of Epsin1 as a regulator for hepatic lipid and glucose metabolism. Genes Dis*.* 2023;10(1):72-75.

2 Huang X, He Q, Zhu H, et al. Hepatic leptin signaling improves hyperglycemia by stimulating MAPK Phosphatase-3 protein degradation via STAT3. Cell Mol Gastroenterol Hepatol*.* 2022;14(5):983-1001.

**Supplementary Data**

**Table S1 Primers for Real-Time quantitative PCR.**

| **Genes** | **Forward** | **Reverse** |
| --- | --- | --- |
| *β-actin* | GGCTGTATTCCCCTCCATCG | CCAGTTGGTAACAATGCCATGT |
| *Glut4* | ACCGGATTCCATCCCACAAG | TCCCAACCATTGAGAAATGATGC |
| *Pparg* | GGAAGACCACTCGCATTCCTT | TCGCACTTTGGTATTCTTGGAG |
| *Pgc1a* | TATGGAGTGACATAGAGTGTGCT | CCACTTCAATCCACCCAGAAAG |
| *Cd36* | ATGGGCTGTGATCGGAACTG | GTCTTCCCAATAAGCATGTCTCC |
| *Dgat1* | TCCGTCCAGGGTGGTAGTG | TGAACAAAGAATCTTGCAGACGA |
| *Fasn* | GGCTCTATGGATTACCCAAGC | CCAGTGTTCGTTCCTCGGA |
| *Scd1* | CCTACGACAAGAACATTCAATCCC | CAGGAACTCAGAAGCCCAAAGC |
| *Acc1* | CGGACCTTTGAAGATTTTGTCAGG | GCTTTATTCTGCTGGGTGAACTCTC |
| *Acc2* | GGAAGCAGGCACACATCAAGA | CGGGAGGAGTTCTGGAAGGA |
| *Srebf1* | AACTGCCCATCCACCGACTC | ATTGATAGAAGACCGGTAGCGC |
| *Hsl* | TGAAGCCAAAGATGAAGTGAGAC | CTTGACTATGGGTGACGTGTAGAG |
| *Ppara* | TACTGCCGTTTTCACAAGTGC | AGGTCGTGTTCACAGGTAAGA |
| *Atgl* | CTGTGTGGAACCAAAGGACCTG | GCTACCCGTCTGCTCTTTCATC |
| *Mgll* | CGGACTTCCAAGTTTTTGTCAGA | GCAGCCACTAGGATGGAGATG |
| *Ucp1* | GCCAAAGTCCGCCTTCAGAT | CAGTTTCGGCAATCCTTCTGTT |
| *Prdm16* | ACAAGTCCTACACGCAGTTCTC | ATGCTGCCAGGCGTGTAATG |
| *Tnfa* | GACCCTCACACTCAGATCATCTTCT | CCACTTGGTGGTTTGCTACGA |
| *Il6* | TAGTCCTTCCTACCCCAATTTCC | TTGGTCCTTAGCCACTCCTTC |
| *F4/80* | TGACTCACCTTGTGGTCCTAA | CTTCCCAGAATCCAGTCTTTCC |
| *Cd11c* | CTGGATAGCCTTTCTTCTGCTG | GCACACTGTGTCCGAACTCA |
| *Mcp1* | TTAAAAACCTGGATCGGAACCAA | GCATTAGCTTCAGATTTACGGGT |
| *Mcp2* | CCCTTCGGGTGCTGAAAAG | CCACTTCTGTGTGGGGTCTAC |


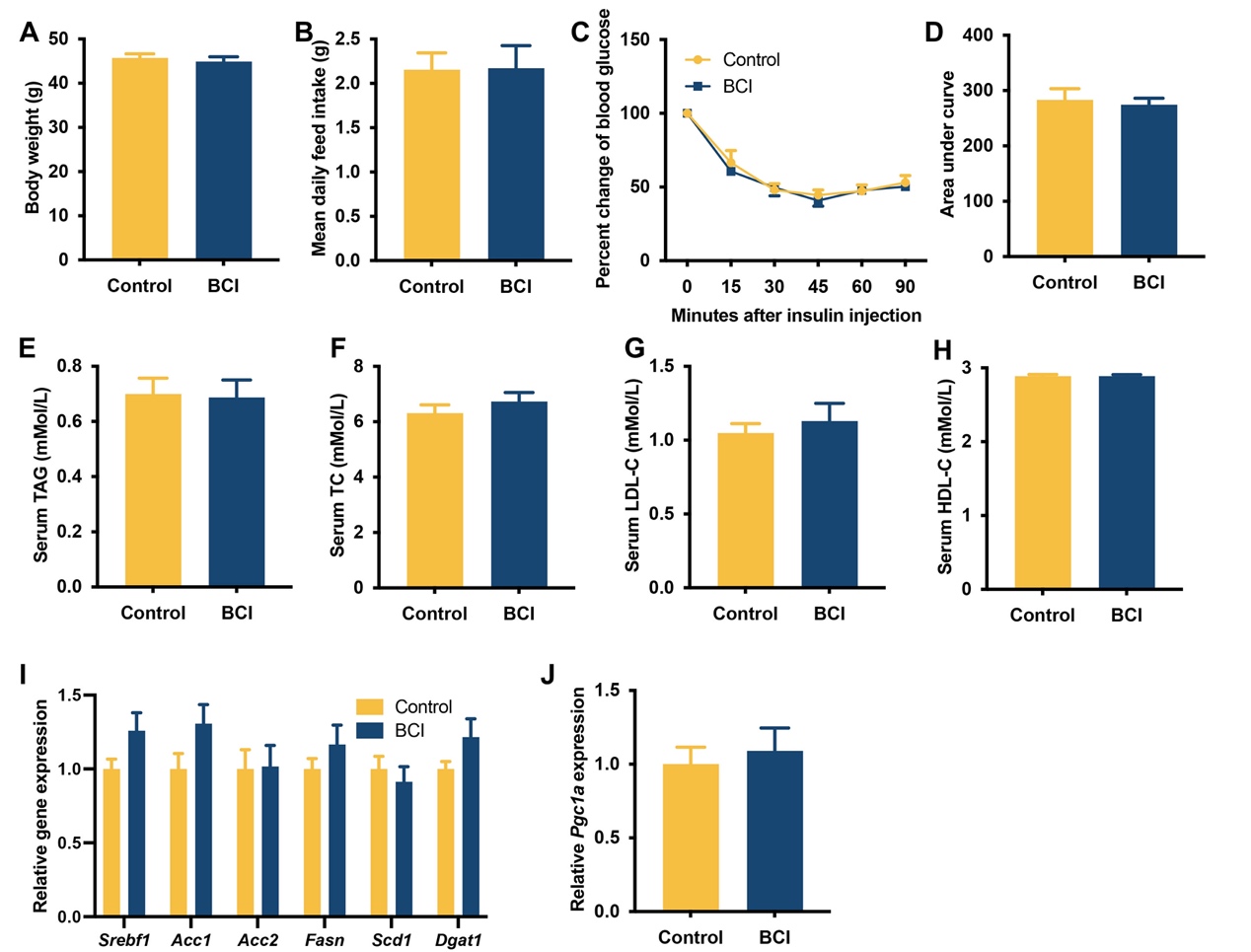


**Figure S1** The role of BCI in the regulation of glucose and lipids metabolism in DIO mice. C57BL/6J mice were fed with a high-fat diet for 16 weeks, and then were injected intraperitoneally with 0.5 mg/kg BCI or vehicle daily for 4 weeks, followed by scarification for serum and tissue collection under fed state (*N* = 6 per group). (A) Body weight. (B) Food intake. 12 mice were housed individually. The initial food weight was recorded per cage at the beginning of the treatment, and the left food weight was measured at the end of the treatment. Food intake were calculated as: (initial food weight - the left food weight) /28 days. (C, D) Insulin tolerance test study (*N* = 6 for each group). (E) TAG content in the serum. (F) TC content in the serum. (G) LDL-C content in the serum. (H) HDL-C content in the serum. (I) Expression levels of lipids synthetic genes in the epididymal adipose tissue. (J) The expression level of *Pgc1a* in the epididymal adipose tissue. Data are expressed as Mean ± SE.


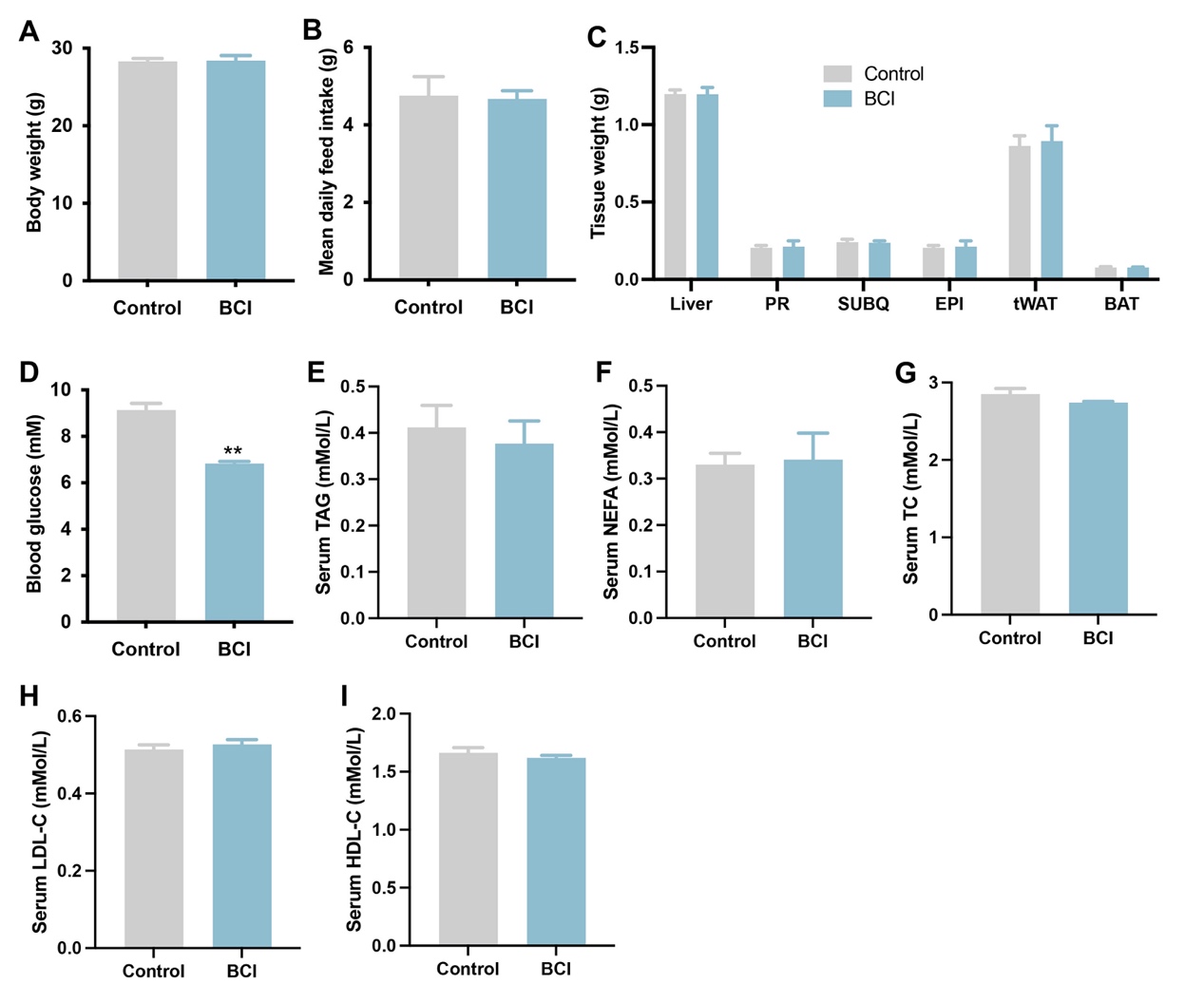


**Figure S2** The role of BCI in the regulation of glucose and lipids metabolism in normal lean mice. C57BL/6J mice were fed with a normal chow diet for 16 weeks, and then were injected intraperitoneally with 0.5 mg/kg BCI or vehicle daily for 4 weeks. Mice were sacrificed for serum and tissue collection under fed state (*N*=3-4 per group). (A) Body weight. (B) Food intake. (C) Tissue weight. (D) Blood glucose level at harvest. (E) TAG content in the serum. (F) NEFA content in the serum. (G) TC content in the serum. (H) LDL-C content in the serum. (I) HDL-C content in the serum. Data are expressed as Mean ± SE. ***P* < 0.01 as compared to control. PR, perirenal adipose tissue; SUBQ, subcutaneous adipose tissue; EPI, epididymal adipose tissue; tWAT, total white adipose tissue; BAT, brown adipose tissue.
